# Women's preferences, impacts, and satisfaction with companion support during labour and delivery experiences in Oman

**DOI:** 10.3389/fgwh.2025.1524270

**Published:** 2025-01-30

**Authors:** Nasar Alwahaibi, Fatema Alajaimi, Hoor Alhabsi, Alzahra Alkalbani, Rodina Aljulandani

**Affiliations:** Department of Biomedical Science, College of Medicine and Health Sciences, Sultan Qaboos University, Muscat, Oman

**Keywords:** companion, childbirth, impacts, preference, pregnant women

## Abstract

**Background:**

This is the first study to combine both the women's preferences, impacts, and satisfaction during their labour and delivery experience and their companions for their role and impacts. Previously published papers examined either the preferences of pregnant women or those of their companions, which are few. Women's preferences, impacts, and satisfaction with their companions during labour and delivery were evaluated in this study.

**Methods:**

This cross-sectional observational study was conducted between June 2022 and April 2024. As part of this study, labouring women as well as their companions were interviewed separately face-to-face in a private place, and all answers were kept anonymous and confidential. We evaluated each category separately based on two separate sections.

**Results:**

This study included a total of 444 labouring women and an equal number of companions, with mean ages of 32.25 years and 42.66 years, respectively. The majority of women and companions were housewives with college degrees. Among companions, mothers and husbands were the most preferred, and they were typically present from admission to discharge. Among women who had companions, 84.1% reported feeling calmer and more comfortable. 91.9% of companions supported women with encouraging words, and 92.6% perceived their support as highly beneficial to the women. Women expressed 94.8% satisfaction with the medical staff and 87.6% satisfaction with hospital services, while companions reported 96.6% and 74.8% satisfaction with the medical staff and hospital services, respectively.

**Conclusions:**

Labouring women appreciated and valued the presence of companions during childbirth as they had positive impacts. Mothers are the most preferred companions and preferred to be present from admission until discharge. Most companions support their labouring women by encouraging wards. Labouring women and companions were satisfied with the medical team and services provided to them. The presence and role of companions during the childbirth process are crucial and warrant emphasis.

## Background

All healthcare systems globally are obligated to offer respectful maternity care to women. The World Health Organization (WHO) highlighted this imperative in 2020 by affirming every woman's right to have a chosen companion during childbirth (1). Several studies have revealed that the absence of companionship during labour can lead to maternal anxiety, prolonged labor, panic, and fear, particularly among first-time mothers (2–4). The fear of childbirth is a common concern among women during this period, with studies reporting rates ranging from 3.7% to 42.4% (5–8). Despite both mother and baby being in good health, fear of childbirth often results in elective cesarean sections (9–11). Additionally, healthcare professionals such as obstetricians, midwives, and nurses also benefit from labour companionship, as it enables them to communicate more effectively with labouring women (12, 13). Despite the acknowledged benefits of labour companionship, its implementation remains inadequate, particularly in developing countries (12, 14). Several barriers hinder the adoption of labour companionship, including limited space in labour and delivery units, the absence of private rooms for women, hospital rules prohibiting companionship (as in Thailand), and societal norms that restrict a woman's choice of companion (15, 16).

Throughout labour and birth, a pregnant woman selects a companion who provides her with continuous physical and emotional support. This study draws on the WHO framework for respectful maternity care, which highlights the importance of supportive care during childbirth (1). Supportive care, in this context, refers to the continuous physical, emotional, and informational assistance provided to women during labour and delivery by a chosen companion, healthcare provider, or both. This care includes verbal and non-verbal communication, advice, or physical help such as massages and touch. There are many positive effects that companions can have during childbirth, including reducing morbidity, pain, labour and delivery duration, and the likelihood of cesarean (17–19). In fact, the presence of a family member or friend during the intrapartum period, even if the person just sits and does nothing, has been reported to improve the experience of intrapartum care for mothers (20). The companion's support includes verbal and non-verbal communication, advice, or physical help such as massages and tough (21).

Sultanate of Oman is located in West Asia and is situated on the southeastern coast of the Arabian Peninsula, and extends to the Persian Gulf mouth at the mouth of the Arabian Sea. As far as the land borders are concerned, Oman shares land borders with Saudi Arabia, the United Arab Emirates, and Yemen, as well as maritime borders with Iran and Pakistan. The capital and largest city is Muscat. There are 5.167,437 million people living in Oman in 2024, including 2,940,271 Omanis, of which 1,460,553 (49.7%) are females (22). The Oman Ministry of Health provides almost free health services to all Omanis, with a small amount of fee (3.12 USD yearly to open a card, and 0.52 USD for each visit), including for antenatal care. According to the available data in 2019, the maternal mortality rate in Oman was 14.1 per 100,000 live births (23). The fertility rate for Oman in 2023 was 2.595 births per woman, and the birth rate was 15.816 births per 1,000 women (24). While Oman has reasonable birth rates and provides assistance to women during childbirth, there is no clear policy about who should provide support during labour and when.

Earlier, we assessed the impact of companions during childbirth based on their perspectives and discovered that 214 women in labour experienced enhanced well-being and calmness due to their companions’ presence (25). Additionally, there has been no previous study to investigate labouring women's preferences, the presence of companions, and their satisfaction with the healthcare services received during childbirth. Therefore, this study aimed to evaluate Omani women's preferences, the impacts of companionship during labour and delivery, and their satisfaction with this aspect of healthcare.

## Methods

### Ethical consideration

This study received approval from the Medical Research Ethics Committee (MREC) at the College of Medicine and Health Sciences, Sultan Qaboos University (SQU), Oman, with the ethical approval number SQU-MREC #2776. Before commencing the study, a comprehensive explanation of the study procedure was provided, and each pregnant woman and companion signed an informed consent form. For illiterate individuals, witnesses who were not part of the research team were asked to witness the entire process and sign the informed consent form. All participants were informed that they had the right to withdraw from the study at any time without being questioned about their decision. It was also clarified that their withdrawal would not affect the medical services provided to them as pregnant women and companions.

### Participants

Sultan Qaboos University Hospital, Muscat, Sultanate of Oman, which established in 1990, is a 700-bed tertiary care teaching hospital. This hospital is a governmental referral university hospital. The facility at the department of Obstetrics and Gynecology includes 9 delivery suite beds, 26 gynecology and antenatal beds, 24 postnatal beds, 2 triage rooms for obstetrical patients and 2 operative rooms for both gynecological and obstetrical cases. According to the last five years data, there are about 3,274 Omani deliveries per year with an average of 273 births per month. Usually, labouring women are accompanied by a companion to the hospital.

### Study design

This cross-sectional observational study took place between June 2022 and April 2024 at the Obstetric ward of Sultan Qaboos University Hospital in Oman. The inclusion criteria involved Omani women of all age groups who had given birth to a live normal baby, as well as companions of various ages, genders, and relationships (mother, father, sister, husband, friend, etc.) who were willing to participate in the study. Exclusion criteria included women with severe illness or medical conditions that could hinder their participation, such as severe postpartum hemorrhage or infection, participants facing communication difficulties due to illness or language barriers, and those who declined to be interviewed.

Each woman and companions were individually interviewed by two research assistants in the postnatal unit following the birth of their baby. The questionnaire was available in both English and Arabic versions, and participants were interviewed face-to-face at a time convenient for them in a private setting to ensure confidentiality. For companions who left the hospital early, a telephone interview was conducted. The questionnaire comprised self-developed questions and others obtained from literature reviews (17, 19, 25). It's important to note that not all interviewed women and companions are necessarily related; some women may participate in the study while their companions decline, and vice versa.

### Sample size calculation

Using the formula $N=p(1-p)z^{2}/d^{2}$ (26), 95% confidence interval, prevalence of 50%, 5% margin of error, and 15% non-response rate (to avoid any incorrectly filled-out questionnaires), the sample size was 442. A pilot study was conducted among 15 women and companions who fulfilled the research criteria. Those who participated in the pilot study were excluded from the study. In addition, five experts reviewed the questionnaire. Some questions were subsequently modified to be understandable.

### Data collection

The questionnaire was divided into two sections, one for labouring women and another for companions. For labouring women, the first section covered details such as age, place of residence, years of marriage, educational level, occupation, financial status, husband's education and occupation, number of children, birth spacing duration, type of newborn, planned pregnancy, history of cesarean deliveries, and overall health status. The second section focused on aspects like the presence and type of companion, timing and duration of companionship, mode of delivery, pregnancy complications, labour and delivery duration, and satisfaction with the medical team and hospital services.

The companion section's first part included information about the companion's age, place of residence, relationship with the labouring woman, educational background, occupation, income, and health status. The second part included the nature of the assistance provided by the supportive companion (such as massage, touch, encouraging words, transfer things, child care), any challenges faced during this assistance and their causes, the impact of the support on the labouring women, and whether the presence of a supportive companion had a positive or negative impact. Additionally, companions were asked about their willingness to support again, whether they would encourage others to be companions, and their satisfaction level with the efforts of the medical team and services provided to them as companions.

### Data analysis

Data was analyzed using IBM SPSS Statistics 29.0 (IBM Corp. Released 2023. IBM SPSS Statistics for Windows, Version 29.0. Armonk, NY: IBM Corp). For the descriptive purposes, categorical data was presented as frequencies and percentages, and continuous data was presented as mean with standard deviation (SD). Associations between categorical variables were assessed using Chi-square test, while associations between continuous and categorical variables were tested using ANOVA test or independent T test, depending on the nature of the studied variables. A *P*-value of <0.05 was considered as statistical significance.

## Results

### Labouring women

A total of 444 labouring women were enrolled in this study. The average age was 32.25 years. The largest proportion come from Muscat (40.8%), followed by Al Batinah South (32.0%). About 54.5% of these women worked as housewives, and 52.7% held college degrees. The majority of their husbands (94.0%) were employed, and 47.5% had completed secondary education. Additionally, most women (78.8%) had previous childbirth experiences, with 51.1% having given birth between two to five years ago. Furthermore, the majority of participants did not have a history of infertility, diabetes, hypertension, previous cesarean sections, or gestational diabetes. 56.98% of those women had planned their pregnancy (Table 1).

**Table 1:** Socio-demographic characteristics of labouring women and associations between variables and the effect of companion presence at Sultan Qaboos University Hospital, Oman, (*n* = 444).

Variables				
Age		32.25 (±5.571)		
No. of years of marriage		7.82 (±5.307)		
No. of children		2.03 (±1.700)		
		Number	Percent	*P*-values
Residence	Muscat	181	40.8	0.207
	Al-Batinah South	142	32.0	
	Al-Batinah North	46	10.4	
	Al-Dakhiliyah	47	10.6	
	Al-Sharqiyah North	15	3.4	
	Al-Sharqiyah South	8	1.8	
	Al-Dhahirah	4	0.9	
	Dhofar	1	0.2	
Educational level	Primary education	1	0.2	0.366
	Preparatory education	17	3.8	
	Secondary education	157	35.4	
	Undergraduate	234	52.7	
	Postgraduate	35	7.9	
Employment status	Housewife	242	54.5	0.002
	Employee	199	44.8	
	Retired	3	0.7	
Husband's educational level	Illiterate	1	0.2	0.843
	Primary education	5	1.1	
	Preparatory education	28	6.3	
	Secondary education	211	47.5	
	Undergraduate	169	38.1	
	Postgraduate	30	6.8	
Husband's employment status	Employee	413	93.0	0.489
	Free Business	17	3.8	
	Retired	12	2.7	
	Does not work	2	0.5	
Spacing period (excluding first-time mothers)	10 months–2 years	93	20.9	0.007
	2–5 years	277	51.1	
	More than 5 years	24	25	
Hypertension	Yes	15	3.4	1.000
	No	429	96.6	
Diabetes	Yes	9	2.0	1.000
	No	435	98.0	
Previous infertility	Yes	9	2.0	0.113
	No	435	98.0	
Gestational diabetes	Yes	142	32.0	0.659
	No	302	68.0	
Previous cesarean section	Yes	60	13.5	0.266
	No	384	86.5	
Type of room	Common	352	79.3	0.115
	Private	92	20.7	
Planned pregnancy	No	191	43.02	0.442
	Yes	253	56.98	

**Figure 1:**
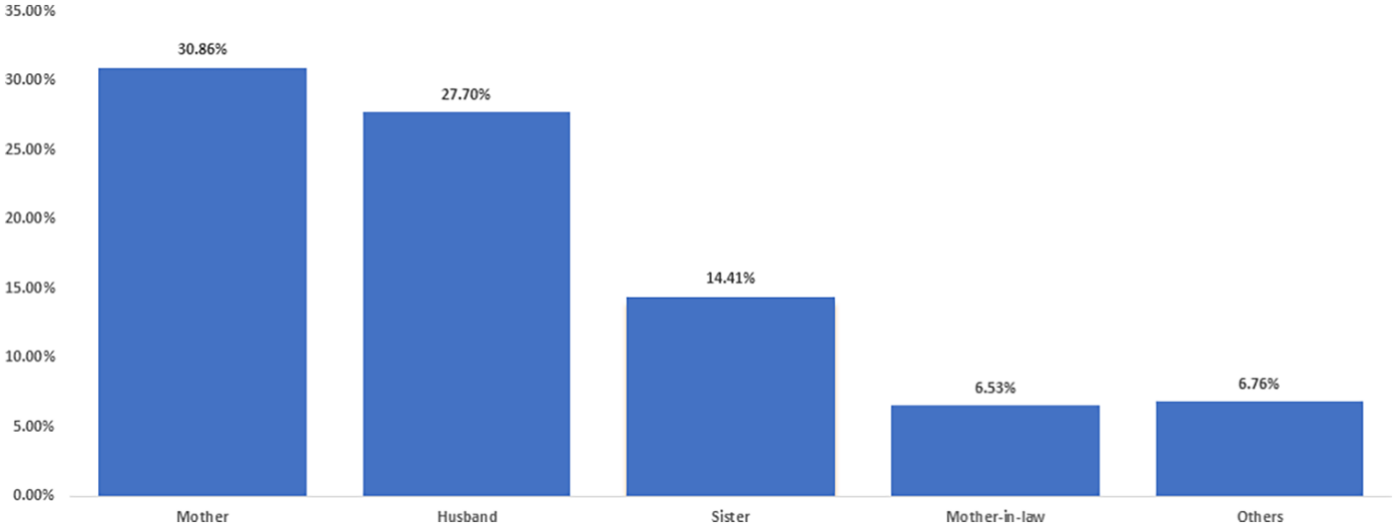
Women's preferred companions during their labor and delivery experience at Sultan Qaboos University Hospital, Oman, (*n* = 444).

Most women (86.26%) indicated a preference for having companions during their labour and delivery, while the remaining women either did not express a preference or were unable to have a companion present (13.74%). Among these preferences, most favored having their mothers present (30.86%), followed by their husbands (27.70%), sisters (14.41%), mother-in-law (6.53%), and other relatives (6.76%) (Figure 1). Additionally, 43.77% of these women indicated a preference for their companions to be present from admission to discharge, whereas 25.59% preferred their companions' presence after childbirth. 14.48% opted for support during the birthing delivery itself. Furthermore, 9.76% of women expressed a preference for companion support during labour pains at the hospital, in contrast to 6.40% who preferred companion support before or upon arriving at the hospital.

Women with companions (*n* = 383) reported significantly higher levels of calmness and comfort during labour, with 84.1% stating they were “a lot calmer and more comfortable” compared to only 19.7% of women without companions (*n* = 61) (*p* < 0.001). On the other hand, 72.1% of women without companions indicated that the presence or absence of a companion did not affect their experience, compared to only 4.9% of those with companions. Satisfaction with medical staff was high across both groups, with 95.3% of women with companions and 91.8% of women without companions reporting satisfaction, although this difference was not statistically significant (*p* = 0.226). Similarly, satisfaction with the services provided was reported by 88.3% of women with companions and 83.6% of those without companions, also with no significant difference (*p* = 0.307). Regarding the nature of birth, the majority of women in both groups had natural births (77.8% with companions vs. 67.2% without companions, *p* = 0.139). The proportion of cesarean sections was slightly higher among women without companions (31.1%) compared to those with companions (21.1%), though this difference was not statistically significant. Other factors, including difficulties during pregnancy, complications after delivery, and the duration of labour and delivery, showed no statistically significant differences between the two groups. However, a slightly higher percentage of women without companions reported complications after delivery (23.0% vs. 17.2%, *p* = 0.280) (Table 2).

**Table 2:** Comparative analysis of birth experiences among women with and without companions during childbirth at Sultan Qaboos University Hospital, Oman, (*n* = 444).

		Have you had a companion during childbirth?		
		Yes (383)	No (61)	*P*-value
Birth experience	I was a lot calmer and more comfortable	84.1%	19.7%	<0.001
	I was a little relaxed and calm	11.0%	8.2%	
	The experience did not differ, whether or not in the presence of a companion	4.9%	72.1%	
Nature of birth	Natural birth	77.8%	67.2%	0.139
	Cesarean section	21.1%	31.1%	
	Vaginal dilation	1.0%	1.6%	
Difficulties during pregnancies	Yes	38.1%	44.3%	0.361
	No	61.9%	55.7%	
Complications after delivery	Yes	17.2%	23.0%	0.280
	No	82.8%	77.0%	
Duration of labour pains	0–12 h	62.9%	68.9%	0.619
	12–24 h	20.6%	18.0%	
	24–48 h	9.7%	4.9%	
	>48 h	6.8%	8.2%	
Duration of delivery in hours	<6 h	83.3%	91.8%	0.290
	6–12 h	9.1%	4.9%	
	>12 h	7.6%	3.3%	
Satisfaction with medical staff	Yes	95.3%	91.8%	0.226
	No	4.7%	8.2%	
Satisfaction with services provided	Yes	88.3%	83.6%	0.307
	No	11.7%	16.4%	

### Companions

A total of 444 companions were included. The mean age of the companions was 42.66 ± 10.12 years. The majority of them were from Muscat (37.6%), followed by South Batinah (32.4%). Majority (40.8%) of companions were mothers, followed by husbands (22.1%). The most common education level was undergraduate (31.8%), followed by secondary (27.5%) and 17.1% were illiterates. Most of the included companions were housewives (47.3%), and 47.1% were workers. The majority (61.9%) of participants have their own cars, and around half (50.7%) have own income. Half of the participants did not employ a housemaid at their houses. Around 15.5% of the participants were diabetics, and 16.0% were hypertensive. Majority (76.8%) of the related patients occupied common room while a minority in a private room (23.2%) (Table 3).

**Table 3:** Socio-demographic characteristics of companions at Sultan Qaboos University Hospital, Oman, (*n* = 444).

Variables	Number (444)	Percent
Age in years (mean ± SD)	42.66 ± 10.12	
Region		
Muscat	167	37.6
South Batinah	144	32.4
Dhakiliyah	58	13.1
North Batinah	33	7.4
North Sharqiya	21	4.7
South Sharqiya	13	2.9
Ad Dhahira	5	1.1
Buraimi	1	0.2
Musandam	1	0.2
Dhofar	1	0.2
Relationship		
Mother	181	40.8
Husband	98	22.1
Sister	45	10.1
Husband sister	87	19.6
Mother-in-Law	15	3.4
Others	18	4.1
Education		
Illiterate	76	17.1
Preparatory	42	9.5
Primary	38	8.6
Secondary	122	27.5
Undergraduate	141	31.8
Postgraduate	25	5.6
Occupation		
Housewife	210	47.3
Working	209	47.1
Not working/Retired	25	5.6
Own car		
No	169	38.1
Yes	275	61.9
Own income		
No	219	49.3
Yes	225	50.7
Housemaid		
No	222	50.0
Yes	222	50.0
Diabetic		
No	375	84.5
Yes	69	15.5
Hypertensive		
No	373	84.0
Yes	71	16.0
Type of room		
Common	341	76.8
Private	103	23.2

The majority (52.5%) of companions provided support from admission to discharge, while 31.3% helped during childbirth. Around 30.2% supported their patients in the immediate post-partum ward, and only 7.7% provided support during birth. The most common type of support provided by companions was encouraging words (91.9%) followed by transferring things (42.8%), massage (31.5%) and touch (26.6%). Only 1.8% provided child care support to their women. Few of them faced problems while helping (6.5%). Most companions (92.6%) perceived their support as very helpful to their women, and only 7.4% and 1.4% of companions reported that they noticed no or little effect, respectively. The vast majority (97.7%) of companions would like to be a companion again, and 98.9% would encourage others to be companions during birth. Most (96.6%) of companions were satisfied with the medical team, and 74.8% were satisfied with the services provided by the hospital (Table 4).

**Table 4:** Analyzing the descriptive statistics of the support outcome variables among companions at Sultan Qaboos University Hospital, Oman, (*n* = 444).

Variables	Number (444)	Percent
When did your help take place?		
From admission to discharge	233	52.5
Before/while arriving at the hospital	55	12.4
During childbirth pain in the hospital	139	31.3
During birth only	34	7.7
Immediately post-partum ward	134	30.2
What is the nature of your help?		
Massage	140	31.5
Touch	118	26.6
Encouraging words	408	91.9
Transfer things	190	42.8
Child care	8	1.8
Did you face any problem in helping?		
No	415	93.5
Yes	29	6.5
What is the effect of your presence as a companion?		
Help her feel comfortable and calm a little	28	6.3
It was reflected positively, which made her feel comfortable and calm	411	92.6
I did not notice any difference	5	1.1
Perceived impact on the women		
No/Small	33	7.4
Good	411	92.6
Will you be a companion again?		
No	10	2.3
Yes	434	97.7
Do you encourage others to be a companion during birth?		
No	5	1.1
Yes	439	98.9
Were you satisfied with the medical team?		
No	15	3.4
Yes	429	96.6
Were you satisfied with services at hospital?		
No	112	25.2
Yes	332	74.8

Transfer things (e.g., bringing essentials like clothing and toiletries, moving personal belongings, or ensuring items are available for mother and baby).

We categorized “It was reflected positively, which made her feel comfortable and calm” as a positive impact, while “helped a little” or “I didn't notice any difference” were grouped as indicating no or small impact. These categories were then compared with other sociodemographic factors. In this context, age of companions, relationship to the patient, and education level of the companions were significantly associated with better perceived impact of the provided support with *P*-values of 0.029, 0.019, and 0.026, respectively. Table 5 shows the detailed results of the relationships between various factors and perceived impact.

**Table 5:** Association between variable factors and the perceived impact of support provided by companions at Sultan Qaboos University Hospital, Oman, (*n* = 444).

Variables	Perceived impact		*P*-value
	No/Small (*n* = 33)	Good (*n* = 411)	
Age in years (mean ± SD)	36.57 ± 7.44	42.95 ± 10.08	0.029
Region			
Muscat	9 (5.4)	158 (94.6)	0.585
Dhakiliyah	2 (3.4)	56 (96.6)	
North Batinah	5 (15.2)	28 (84.8)	
South Batinah	12 (8.3)	132 (91.7)	
Ad Dhahira	1 (20.0)	4 (80.0)	
North Sharqiya	2 (9.5)	19 (90.5)	
South Sharqiya	2 (15.4)	11 (84.6)	
Buraimi	–	1 (100.0)	
Musandam	–	1 (100.0)	
Dhofar	–	1 (100.0)	
Relationship			
Mother	13 (7.2)	168 (92.8)	0.019
Husband	7 (7.1)	91 (92.9)	
Sister	–	45 (100.0)	
Husband's sister	12 (13.8)	75 (86.2)	
Mother-in-law	–	15 (100.0)	
Others	1 (5.6)	17 (94.4)	
Education			
Illiterate	2 (2.6)	74 (97.4)	0.026
Preparatory	4 (9.5)	38 (90.5)	
Primary	2 (5.3)	36 (94.7)	
Secondary	16 (13.1)	106 (86.9)	
Undergraduate	9 (6.4)	132 (93.6)	
Postgraduate	–	25 (100.0)	
Occupation			
Housewife	11 (5.2)	199 (94.8)	0.137
Working	21 (10.0)	188 (90.0)	
Not working/Retired	1 (4.0)	24 (96.0)	
Own car			
No	8 (4.7)	161 (95.3)	0.097
Yes	25 (9.1)	250 (90.9)	
Own income			
No	16 (7.3)	203 (92.7)	1.000
Yes	17 (7.6)	208 (92.4)	
Housemaid			
No	20 (9.0)	202 (91.0)	0.278
Yes	13 (5.9)	209 (94.1)	
Diabetic			
No	29 (7.7)	346 (92.3)	0.803
Yes	4 (5.8)	65 (94.2)	
Hypertensive			
No	30 (8.0)	343 (92.0)	0.331
Yes	3 (4.2)	68 (95.8)	
Type of room			
Common	27 (7.9)	314 (92.1)	0.668
Private	6 (5.8)	97 (94.2)	

In addition, we explored if support related characteristics can affect the perceived impact. There was significant association between perceived impact of help and whether the companion encourage others to be companions with their women. In this regard, 92.9% of those who encourage other perceived their support to be of a good effect, compared to 60% among those who do not encourage (*P*-value 0.047). Table 6 gives the details of the association results between support characteristics and perceived impact.

**Table 6:** Association between characteristics related to support and the perceived impact of the support provided by companions at Sultan Qaboos University Hospital, Oman, (*n* = 444).

Variables	Perceived impact		*P*-value
	No/Small (*n* = 33)	Good (*n* = 411)	
When did your help take place?			
From admission to discharge	15 (6.4)	218 (93.6)	0.470
Before/while arriving at the hospital	6 (10.9)	49 (89.1)	0.277
During childbirth pain at the hospital	8 (5.8)	131 (94.2)	0.438
During delivery only	4 (11.8)	30 (88.2)	0.304
Immediate post-partum ward	8 (6.0)	126 (94.0)	0.556
What is the nature of your help?			
Massage	12 (8.6)	128 (91.4)	0.561
Touch	9 (7.6)	109 (92.4)	1.000
Encouraging words	30 (7.4)	378 (92.6)	0.742
Transfer things	21 (11.1)	169 (88.9)	0.016
Childcare	2 (18.2)	9 (81.8)	0.193
Did you face any problem in helping?			
No	31 (7.5)	384 (92.5)	1.000
Yes	2 (6.9)	27 (93.1)	
Will you be a companion again?			
No	2 (20.0)	8 (80.0)	0.166
Yes	31 (7.1)	403 (92.9)	
Do you encourage others to be a companion during birth?			
No	2 (40.0)	3 (60.0)	0.047
Yes	31 (7.1)	408 (92.9)	
Were you satisfied with the medical team?			
No	–	15 (100.0)	0.616
Yes	33 (7.7)	396 (92.3)	
Were you satisfied with services at hospital?			
No	6 (5.4)	106 (94.6)	0.409
Yes	27 (8.1)	305 (91.9)	

## Discussion

Previous studies have typically focused on either the preferences of pregnant women or those of their companions, with relatively few addressing both. To our knowledge, this is one of the few studies to examine women's preferences, impacts, and satisfaction during labour and delivery alongside the roles and effects of their companions.

The present study shows that the percentage of the women who had companions during their childbirth was 86.26%. Our findings demonstrate that birth companions play a crucial role in improving the labour and delivery experience for women in Oman. Anchoring this study in the supportive care model highlights the emotional, physical, and informational support companions provide, which aligns with patient-centered care initiatives in maternal health.

The high percentage of women who preferred the presence of a companion during delivery indicates that they are aware of the importance of receiving assistance during such a challenging time. This finding is much higher than a study conducted in Saudi Arabia where only 45.3% of the Saudi women preferred companions during childbirth (27). In addition, another recent study in Saudi Arabia reported that only 43.2% of labouring women had companion present during delivery (28). Furthermore, our finding is higher than a similar study in the United Arab Emirates, where 77% of labouring women preferred the presence of support during childbirth (17).

Mothers are the most preferred companions during labour and delivery with the Omani women with 30.86%. In general, the daughters are very close to their mothers, they feel more comfortable and safer with them, and they feel loved by them (25). In addition, mothers who have been through this experience have a deep understanding of what their daughters are experiencing. This finding is in line with another study where 418 Tanzanian women reported that mothers (34.5%) were the most common companions during childbirth (12). Similarly, another two studies conducted in Saudi Arabia and United Arab Emirates reported that mothers with 58% and 59% were the most preferred companions during childbirths, respectively (17, 27). Women often prefer their mothers as birth companions because of cultural norms and trust in their experience with childbirth. This choice provides a sense of calm and comfort during labour. In addition, the encouragement and constant presence of companions were seen as very helpful by both women and their companions, reinforcing the idea that companionship reduces stress and improves emotional well-being.

Omani women in this study reported that the presence of companions during their childbirths made them calmer and more comfortable (84.1%). This high percentage reflects the important role that the companions play during the labour and delivery. In agreement with this finding, women in Poland, Brazil, and Iran felt better and calmer having had their companions with them during labour and delivery (29–31). Labouring women preferred their companions to be present from the moment of arrival to the hospital until discharge as represented by 43.77%. This desire shows the positive effects on the women during their labour and delivery. In agreement with this finding, Brazilian women showed that the moments of companionship were 74.8% during labour and delivery (19).

Interestingly, 56.98% of the Omani women planned for their pregnancy and 51.1% had a birth spacing period between two and five years. This finding suggests that a significant number of married couples use a family planning. Family planning has been discussed mainly in developing countries as women are interested in education and work (32). It has been reported that family planning leads to improvements in health, social, and well-being (33). Another interesting finding in this study was that 32.0% of Omani women have the gestational diabetes mellitus (GDM). The average age of the surveyed women was 32 years old, which is highly associated with the GDM (34). The prevalence of previous cesarean in this study was 13.5%, which falls within the recommended rate of worldwide all cesarean deliveries (10%–15%) set by the WHO (35). According to a previous study conducted at Sultan Qaboos University Hospital, Oman, over a period of three years (1998–2001) to review caesarean sections, the rate was 13% (36), which is similar to our finding. Globally, other countries reported different prevalence rates of cesarean deliveries, Saudi Arabia (32.6%), USA (14.2%), and Iran (47.9%) (37–39).

The present study reveals high levels of satisfaction among Omani women with the care they received during childbirth. Specifically, 94.8% of women with companions reported being satisfied with the medical staff compared to 91.8% of those without companions. Similarly, 88.3% of women with companions were satisfied with the medical services provided, compared to 83.6% of those without companions, although these differences were not statistically significant. These findings underscore the overall quality of care provided to labouring women at the hospital and highlight the potential role of companions in enhancing perceptions of care. In line with this finding, companions themselves reported 96.6% and 74.8% satisfaction with the medical staff and services, respectively, further reflecting the hospital's commitment to addressing the needs of both labouring women and their support persons. In agreement with this finding, the majority of women in Saudi Arabia (75%) were satisfied with the medical care provided to them (27). Similarly, the women in Malawi, Ethiopia, and Iran were satisfied with the care they received during their childbirths with 97.3%, 90.2%, and 59.5%, respectively (40–42).

The presence of birth companions has been associated with fostering respectful maternity care and mitigating mistreatment of women during childbirth. Companions act as supporters for labouring women, providing emotional support and ensuring that their dignity is maintained. This aligns with the human rights-based approach to maternity care, which highlights the importance of supportive practices to reduce mistreatment and enhance women's childbirth experiences. In this study, many women expressed high levels of satisfaction with the medical staff (94.8%), and we hypothesize that the presence of companions contributed to this positive perception. Companions not only provide emotional reassurance but also serve as a source of accountability, encouraging respectful and patient-centered interactions between women and healthcare providers. While our study did not directly investigate mistreatment, these findings suggest that the presence of companions may play a protective role in promoting respectful care and improving overall satisfaction.

From the companion's point of view, the present study showed that 91.9% of the companions used encouraging words as a help to their labouring women. There is no doubt that encouraging words during childbirth is an easy and effective way for the companions to assist. In addition, 42.8% of companions reported “transferring things” as part of their support. This includes practical tasks such as bringing essential items (clothing, toiletries) to the labour room, moving personal belongings within the hospital, or ensuring that necessary items were available for the mother and baby during their stay. These logistical tasks complement the emotional support provided, reflecting the multifaceted role of companions in addressing both the practical and psychological needs of labouring women. It is also worth noting that this finding is in accordance with another study which showed that 82% of Brazilian companions (52/62) would use encouraging wards as psychological support for their labouring women (18). The results indicate that 52.5% of companions were present from admission to discharge, underscoring their continuous support during labour and delivery. In addition, 31.3% of companions were present specifically during childbirth pain in the hospital, providing emotional and practical assistance to labouring women during this critical phase. Furthermore, 30.2% of companions were present in the immediate postpartum ward, reflecting a preference for offering support during the recovery period and assisting with newborn care. The preferences expressed by labouring women further clarify these patterns. A significant proportion (43.77%) preferred their companions to be present from admission to discharge, highlighting the importance of consistent support. However, 25.59% of women preferred their companions' presence after childbirth, focusing on the postpartum recovery period. In accordance with this finding, 74.8% of Brazilian companions were present during labour and delivery (19). According to the results of this study, 92.6% of companions reported that the women felt calmer and better as a result of their presence. Several studies reported similar findings (19, 25, 29, 30, 31). Additionally, 97.7% of the companions who took part in the survey said that they would love to accompany their labouring women again, and 98.9% said that they would encourage other companions to assist their labouring women during their labour and delivery.

## Study strengths and limitations

To the best of our knowledge, this is one of the few studies that evaluates both, the women's preferences, impacts, and satisfaction during their labour and delivery experience and their companions for their roles and effects. However, some limitations should be noted. First, the surveyed labouring women and companions were from one single government hospital, those in other governmental and private hospitals were not included. Second, when all postpartum women and their companions provided their responses, it was possible that a recall bias or a fear of service deprivation could have been introduced.

## Conclusion

Labouring women appreciated and valued the presence of companions during childbirth as they had positive impacts. Mothers are the most preferred companions and preferred to be present from admission until discharge. Most companions support their labouring women by encouraging words. Labouring women and companions were satisfied with the medical team and services provided to them. The presence and role of companions during the childbirth process are crucial and warrant emphasis.

## Data Availability

The original contributions presented in the study are included in the article/Supplementary Material, further inquiries can be directed to the corresponding author.

## References

[1] World Health Organization. Available online at: https://www.who.int/news/item/09-09-2020-every-woman-s-right-to-a-companion-of-choice-during-childbirth (accessed March 17, 2024).

[2] Campero L García C Díaz C Ortiz O Reynoso S Langer A. “Alone, I wouldn’t have known what to do”: a qualitative study on social support during labor and delivery in Mexico. Soc Sci Med. (1998) 47:395–403. 10.1016/S0277-9536(98)00077-X 9681909

[3] Sapkota S Kobayashi T Kakehashi M Baral G Yoshida I. In the Nepalese context, can a husband’s attendance during childbirth help his wife feel more in control of labour? BMC Pregnancy Childbirth. (2012) 14(12):49. 10.1186/1471-2393-12-49 PMC3464724 22698006

[4] Stepowicz A Wencka B Bieńkiewicz J Horzelski W Grzesiak M. Stress and anxiety levels in pregnant and post-partum women during the COVID-19 pandemic. Int J Environ Res Public Health. (2020) 17(24):1–9. 10.3390/ijerph17249450 PMC7766953 33348568

[5] Gökçe İsbir G Serçekuş P Yenal K Okumuş H Durgun Ozan Y Karabulut Ö The prevalence and associated factors of fear of childbirth among Turkish pregnant women. J Reprod Infant Psychol. (2024) 42(1):62–77. 10.1080/02646838.2022.2057938 35345941

[6] O’Connell MA Leahy-Warren P Khashan AS Kenny LC O’Neill SM. Worldwide prevalence of tocophobia in pregnant women: systematic review and meta-analysis. Acta Obstet Gynecol Scand. (2017) 96(8):907–20. 10.1111/aogs.13138 28369672

[7] Lukasse M Schei B Ryding EL, Bidens Study Group. Prevalence and associated factors of fear of childbirth in six European countries. Sex Reprod Healthc. (2014) 5(3):99–106. 10.1016/j.srhc.2014.06.007 25200969

[8] Toohill J Fenwick J Gamble J Creedy DK. Prevalence of childbirth fear in an Australian sample of pregnant women. BMC Pregnancy Childbirth. (2014) 14:275. 10.1186/1471-2393-14-275 25123448 PMC4138382

[9] Kabakian-Khasholian T. My pain was stronger than my happiness: experiences of caesarean births from Lebanon. Midwifery. (2013) 29(11):1251–56. 10.1016/j.midw.2012.09.001 23415357

[10] Demšar K Svetina M Verdenik I Tul N Blickstein I Globevnik Velikonja V. Tokophobia (fear of childbirth): prevalence and risk factors. J Perinat Med. (2018) 46(2):151–4. 10.1515/jpm-2016-0282 28379837

[11] El-Aziz SNA Mansour SES Hassan NF. Factors associated with fear of childbirth: it’s effect on women’s preference for elective cesarean section. J Nurs Educ Pract. (2017) 7(1):133–45. 10.5430/jnep.v7n1p133

[12] Dynes MM Binzen S Twentyman E Nguyen H Lobis S Mwakatundu N Client and provider factors associated with companionship during labor and birth in Kigoma region, Tanzania. Midwifery. (2019) 69:92–101. 10.1016/j.midw.2018.11.002 30453122 PMC11019777

[13] Bohren MA Berger BO Munthe-Kaas H Tunçalp Ö. Perceptions and experiences of labour companionship: a qualitative evidence synthesis. Cochrane Database Syst Rev. (2019) 209:120. 10.1002/14651858.CD012449.pub2 PMC6422112 30883666

[14] Bohren MA Hazfiarini A Vazquez MC Colomar M De Mucio B Tunçalp O From global recommendations to (in) action: a scoping review of the coverage of companion of choice for women during labour and birth. Plos Glob Public Health. (2023) 3(2):1–19. 10.1371/journal.pgph.0001476 PMC10021298 36963069

[15] Yaya Bocoum F Kabore CP Barro S Zerbo R Tiendrebeogo S Hanson C Women’s and health providers’ perceptions of companionship during labor and childbirth: a formative study for the implementation of WHO companionship model in Burkina Faso. Reprod Health. (2023) 20(1):46. 10.1186/s12978-023-01597-w 36941676 PMC10029160

[16] Chunuan S Somsap Y Pinjaroen S Thitimapong S Nangham S Ongpalanupat F. Effect of the presence of family members, during the first stage of labor, on childbirth outcomes in a provincial hospital in Songkhla province, Thailand. Pac Rim Int J Nurs Res. (2013) 13(1):16–27.

[17] Mosallam M Rizk DE Thomas L Ezimokhai M. Women’s attitudes towards psychosocial support in labour in United Arab Emirates. Arch Gynecol Obstet. (2004) 269:181–7. 10.1007/s00404-002-0448-7 12748868

[18] Oliveira AS Damasceno AK Moraes JL Moreira K Teles MR Gomes LF. Technology used by companions in labor and childbirth: a descriptive study. Online Braz J Nurs. (2014) 13(1):36–45.

[19] Diniz CS d'Orsi E Domingues RM Torres JA Dias MA Schneck CA Implementation of the presence of companions during hospital admission for childbirth: data from the birth in Brazil national survey. Cad Saude Publica. (2014) 30(Suppl 1):1–14. 10.1590/0102-311X00127013 25167174

[20] Lavender T Walkinshaw SA Walton I. A prospective study of women’s views of factors contributing to a positive birth experience. Midwifery. (1999) 15:40–6. 10.1016/S0266-6138(99)90036-0 10373872

[21] Campos-Garzón C Riquelme-Gallego B de la Torre-Luque A Caparrós-González RA. Psychological impact of the COVID-19 pandemic on pregnant women: a scoping review. Behav Sci (Basel). (2021) 11(12):181. 10.3390/bs11120181 34940116 PMC8698569

[22] The National Center for Statistics and Information, Oman. (2024). Available online at: https://www.ncsi.gov.om/Pages/NCSI.aspx (accessed March 16, 2024).

[23] Ministry of Health. Annual Health Report (2021) Available online at: https://www.moh.gov.om/en/web/statistics/-/-2-10 (accessed February 19, 2024).

[24] Oman Birth Rate 1950-2024 (2024). Available online at: https://www.macrotrends.net/countries/OMN/oman/birth-rate (accessed March 05, 2024).

[25] Alwahaibi N AL-Julandani R Al-Kalbani A. The role and effect of companions during childbirth in Oman. BMC Pregnancy Childbirth. (2024) 24:47. 10.1186/s12884-024-06256-x 38195477 PMC10775649

[26] Pourhoseingholi MA Vahedi M Rahimzadeh M. Sample size calculation in medical studies. Gastroenterol Hepatol Bed Bench. (2013) 6(1):14–7. 24834239 PMC4017493

[27] Al-Mandeel HM Almufleh AS Al-Damri AJ Al-Bassam DA Hajr EA Bedaiwi NA Saudi women acceptance and attitudes towards companion support during labor: should we implement an antenatal awareness program? Ann Saudi Med. (2013) 33(1):28–33. 10.5144/0256-4947.2013.28 23458937 PMC6078577

[28] Mousa O Salameh B Alqahtani M David M Almefarfesh A Alduhilan D Women’s attitudes, prevalence, related factors, and perceived barriers of birth companionship in Saudi Arabia. Women’s Health. (2024) 20. 10.1177/17455057231224553 PMC10822074 38279816

[29] Swiatkowska-Freund M Kawiak D Preis K. Advantages of father’s assistance at the delivery. Ginekol Pol. (2007) 78(6):476–8. 17899705

[30] Bruggemann OM Parpinelli MA Osis MJ Cecatti G Carvalhinho AS. Support to woman by a companion of her choice during childbirth: a randomized controlled trial. Reprod Health. (2007) 4:5. 10.1186/1742-4755-4-5 17612408 PMC1936417

[31] Salehi A Fahami F Beigi M. The effect of presence of trained husbands beside their wives during childbirth on women’s anxiety. Iran J Nurs Midwifery Res. (2016) 21(6):611–5. 10.4103/1735-9066.197672 28194202 PMC5301069

[32] Al-Nahedh NNA. The effect of sociodemographic variables on child-spacing in rural Saudi Arabia. East Mediterr Health J. (1999) 5:136–40. 10.26719/1999.5.1.136 10793791

[33] Utoo BT Mutihir TJ Utoo PM. Knowledge, attitude and practice of family planning methods among women attending antenatal clinic in Jos, North-central Nigeria. Niger J Med. (2010) 19:214–8. 10.4314/njm.v19i2.56524 20642092

[34] American Diabetes Association. Classification and diagnosis of diabetes: standards of medical care in diabetes-2019. Diabetes Care. (2019) 42(Suppl 1):S13–28. 30559228 10.2337/dc19-S002

[35] Betran AP Torloni MR Zhang JJ Gülmezoglu AM. WHO statement on caesarean section rates. BJOG. (2016) 123:667–70. 10.1111/1471-0528.13526 26681211 PMC5034743

[36] Mathew M Kumari R Vaclavinkova V Krolikowski A. Caesarean sections at Sultan Qaboos University Hospital: a three-year review. J Sci Res Med Sci. (2002) 4(1–2):29–32. 24019723 PMC3177246

[37] Alabdullah HA Ismael L Alshehri LA Alqahtani S Alomari M Alammar A The prevalence of c-section delivery and its associated factors among Saudi women attending different clinics of King Khalid University Hospital. Cureus. (2021) 13(1):e12774. 10.7759/cureus.12774 33643728 PMC7885733

[38] Witt WP Wisk LE Cheng ER Mandell K Chatterjee D Wakeel F Determinants of cesarean delivery in the US: a life course approach. Matern Child Health J. (2015) 19(1):84–93. 10.1007/s10995-014-1498-8 24770955 PMC4209310

[39] Shirzad M Shakibazadeh E Hajimiri K Betran AP Jahanfar S Bohren MA Prevalence of and reasons for women’s, family members’, and health professionals’ preferences for cesarean section in Iran: a mixed-methods systematic review. Reprod Health. (2021) 18(1):3. 10.1186/s12978-020-01047-x 33388072 PMC7778821

[40] Changole J Bandawe C Makanani B Nkanaunena K Taulo F Malunga E Patients’ satisfaction with reproductive health services at Gogo Chatinkha Maternity Unit, Queen Elizabeth Central Hospital, Blantyre, Malawi. Malawi Med J. (2010) 22(1):5–9. 10.4314/mmj.v22i1.55899 21618840 PMC3345683

[41] Dewana Z Fikadu T Mariam A Abdulahi M. Client perspective assessment of women’s satisfaction towards labour and delivery care service in public health facilities at Arba Minch town and the surrounding district, Gamo Gofa zone, south Ethiopia. Reprod Health. (2016) 13:11. 10.1186/s12978-016-0125-0 26867797 PMC4750206

[42] Taghavi S Ghojazadeh M Azami-Aghdash S Alikhah H Bakhtiarzadeh K Azami A Assessment of mothers’ satisfaction with the care of maternal care in specialized educational-medical centers in obstetrics and gynaecological disease in northwest, Iran. J Anal Res Clin Med. (2015) 3(2):77–86. 10.15171/jarcm.2015.012

